# Survival outcomes and the prognostic significance of clinicopathological features in patients with endometrial clear cell carcinoma: a 35-year single-center retrospective study

**DOI:** 10.1186/s12957-023-02992-0

**Published:** 2023-03-27

**Authors:** Xiao Ma, Dongyan Cao, Huimei Zhou, Tao Wang, Jinhui Wang, Ying Zhang, Mei Yu, Ninghai Cheng, Peng Peng, Jiaxin Yang, Huifang Huang, Keng Shen

**Affiliations:** National Clinical Research Center for Obstetric & Gynecologic Diseases, Department of Obstetrics and Gynecology, Peking Union Medical College Hospital, Chinese Academy of Medical Sciences & Peking Union Medical College, Peking Union Medical College Hospital (Dongdan Campus), No.1 Shuaifuyuan Wangfujing Dongcheng District, Beijing, 100730 China

**Keywords:** Endometrial neoplasms, Clear cell adenocarcinoma, Mixed endometrial carcinomas, Endometrioid, Serous adenocarcinoma, Prognosis, Surgical pathology

## Abstract

**Background:**

To evaluate the oncological outcomes and the impact of clinicopathological factors on endometrial clear cell carcinoma (ECCC) outcomes.

**Methods:**

Medical records of patients with primary ECCC treated at our center between 1985 and December 2020 were reviewed. Overall survival (OS) and progression-free survival (PFS) were the endpoints. The Kaplan–Meier method and Cox regression analysis were used.

**Results:**

In total, 156 patients were included, of whom 59% and 41% had early- and advanced-stage ECCC, respectively. The median age of onset was 61 years, and 80.8% of the patients were postmenopausal. Ninety-two (59%) and 64 (41%) patients had pure ECCC and mixed endometrial carcinoma with clear cell carcinoma (CCC) components, respectively. Mixed pathological components, elevated cancer antigen 125 levels, positive lymphovascular space invasion, deep myometrial invasion, and malignant peritoneal washing cytology (PWC) were more frequently observed in the advanced stage. Thirty-nine patients (25%) experienced relapse and 32 patients (20.5%) died. The 5-year PFS and OS rates for the entire cohort were 72.6% and 79%, respectively. Multivariate analysis showed that advanced-stage disease and positive PWC significantly decreased PFS, while advanced-stage disease and older age (> 61 years) significantly decreased OS.

**Conclusions:**

ECCC is a rare and aggressive type II endometrial carcinoma that is common in older women and patients with advanced-stage disease. Positive PWC was associated with decreased PFS, although its presence did not influence the stage. Positive PWC, and advanced stage and older age were independent negative prognostic factors.

## Introduction

Endometrial carcinoma (EC) is one of the most common gynecological malignant tumors, surpassed only by cervical cancer, according to the latest data provided by the International Agency for Research on Cancer in 2020 [1]. In the United States, 65,950 individuals will be newly diagnosed with EC by 2022, of whom 12,550 will die of it [2]. Approximately 84,520 new cancer cases and 17,543 deaths due to EC were reported [3].

According to the classical dualistic model introduced by Bokhman [4], EC is generally classified as type I (endometrioid) and type II (non-endometrioid), representing up to 80 and 10% of the cases, respectively [5]. The remaining 10% is classified as mixed endometrial carcinoma (MEC) [5]. Unlike type I EC, type II EC is estrogen-independent, predominantly developing in older women and exhibiting more aggressive characteristics [6]. Endometrial clear cell carcinoma (ECCC) is a distinct subtype of EC that is clinically aggressive and accounts for 1–6% of all ECs [7]. Compared to endometrioid cancer (EmC), ECCC has a higher recurrence rate and worse prognosis.

Although type II EC has a worse prognosis, most studies evaluating the prognosis of these neoplasms have included other histotypes of type II EC, such as serous carcinoma (SC) and MEC. Therefore, a separate assessment of the ECCC outcomes is required. Additionally, the knowledge of independent prognostic factors for ECCC is limited. Hence, our study aimed to evaluate the prognosis of patients with ECCC in an experienced tertiary center and explore the effect of clinicopathological factors on ECCC outcomes.

## Materials and methods

### Patient cohort

The study was approved by the Institutional Review Board of Peking Union Medical College Hospital, and the requirement for informed consent was waived in accordance with the Declaration of Helsinki. Patients diagnosed with primary ECCC between 1985 and December 2020 were identified from our institution’s electronic medical database. Demographic, clinicopathological, treatment modality, and prognostic data were reviewed and retrieved from medical records and via telephone interviews. The histological slides were reviewed by two specialized gynecologic oncology pathologists.

The histological type was determined based on the pathological examination of the uterus. ECCC was diagnosed according to the 2020 World Health Organization Classification of Endometrial Cancer [8]. ECCC is defined as a histotype of type II EC and is characterized by papillary, tubulocystic, and/or solid architectural patterns and variable pleomorphic polygonal, cuboidal, flat, or hobnail cells with a clear or eosinophilic cytoplasm [8]. MEC are carcinomas with two or more discrete histological types, of which at least one component is either SC or clear cell carcinoma (CCC) [8]. The inclusion criteria were as follows: (1) patients pathologically diagnosed with ECCC (pure or mixed) and (2) patients who underwent surgical staging (comprehensive or incomplete). The exclusion criteria were as follows: (1) patients with non-primary ECCC, (2) patients who did not undergo either comprehensive staging or incomplete staging, (3) patients whose detailed medical records were unavailable, and (4) patients who were lost to follow-up.

### Treatment

Total hysterectomy and bilateral salpingo-oophorectomy (THBSO) in addition to surgical staging, are the recommended primary treatments for patients with uterine-confined EC unless they have a strong desire to maintain their childbearing potential. Total hysterectomy (TH), radical hysterectomy plus bilateral salpingo-oophorectomy (BSO), and staging are recommended for patients with suspectedcervical involvement. In addition to hysterectomy and bilateral adnexectomy, comprehensive staging of patients with ECCC also involves peritoneal washing, pelvic and para-aortic evaluation, omental biopsy or omentectomy, and biopsy of suspicious lesions [9]. In patients with suspected extrauterine disease, maximum cytoreductive efforts should be made to eradicate any visible and measurable lesions [9]. Optimal cytoreduction was defined as a residual lesion measuring ≤ 1 cm. Surgical pathological staging was performed according to the International Federation of Gynecology and Obstetrics (FIGO) 2009 classification [10], and those diagnosed before 2009 were re-staged. The patients underwent either observation alone or adjuvant therapy following surgery. Adjuvant therapy includes chemotherapy (CT), radiation therapy (RT), and chemoradiation (CRT).

### Follow-up

At the end of the primary treatment, patients were followed-up according to the following plan: every 3 months for 2–3 years, every 6 months for 5 years, and annually thereafter. Follow-up evaluations included gynecological examination, pelvic and abdominal ultrasound, and serum cancer antigen 125 (CA125) test. Computed tomography, magnetic resonance imaging, or positron emission tomography-computed tomography was performed when metastases were suspected.

### Outcome

The medical records of all patients were reviewed from the date of diagnosis to the date of disease-related death or end of the follow-up period (June 10, 2022). We aimed to examine the survival outcomes of patients with ECCC and identify the clinicopathological factors that are correlated with worse outcomes. Overall survival (OS) was calculated from the date of the primary surgery to the date of disease-related death or last follow-up. Progression-free survival (PFS) was calculated from the date of the primary surgery to the date of recurrence, progression, or last follow-up.

### Statistics

Descriptive statistics was used to describe patient characteristics. Continuous variables were expressed as mean ± standard deviation or median (range) and assessed using the Shapiro–Wilk test to determine their distribution, while categorical data were expressed as numbers (percentages). Student’s t-test and Mann–Whitney U test were used to analyze continuous variables. Pearson’s chi-square and Fisher’s exact tests were used to analyze categorical variables. The reverse Kaplan–Meier method was used to calculate the median follow-up time and 95% confidence intervals (CI). Survival curves for PFS and OS were constructed using the Kaplan–Meier method with the log-rank test. Patients with no survival events at the last contact were censored. A Cox proportional hazard regression model was used to determine potential prognostic factors for PFS and OS. Multivariate analysis was performed with the inclusion of covariates with a *p* value of < 0.1 in the univariate analysis. Statistical analyses were performed using SPSS (version 25.0; SPSS Inc., Chicago, IL, USA) and GraphPad Prism software (version 7.0). Statistical tests were two-tailed, and a *p* value < 0.05 was considered significant.

## Results

### Patient demographics

**Fig.1:**
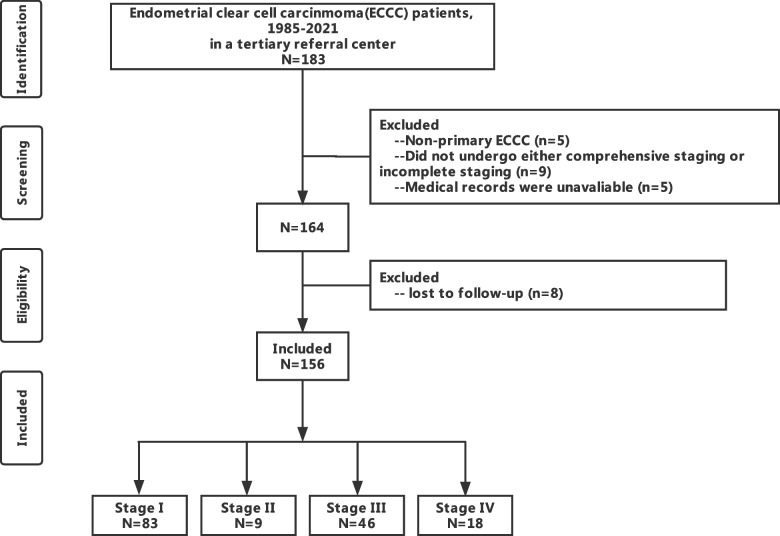
The flow diagram of the study

A flow diagram of this study is shown in Fig. 1. A total of 183 patients with ECCC were identified between 1985 and December 2020. Based on the inclusion and exclusion criteria, 156 patients were included in this study and their medical data were analyzed. Demographic and clinicopathological features are summarized in Table 1. The median age at diagnosis was 61 years (range, 31–86 years), and 80.8% of patients were menopausal. The median serum CA125 level was 22.1 IU/mL (range, 5–736.2); about one-third of the patients had elevated CA125 levels.

**Table 1:** Demographic and morphological features

**Age at diagnosis, median (range), years**	61 (31–86)
**Menopause**	
Yes	80.8% (126/156)
No	19.2% (30/156)
**Medical comorbidities**	
Hypertension	36.5% (57/156)
Diabetes	21.2% (33/156)
History of Colorectal cancer	5.1% (8/156)
History of Breast cancer	3.2% (5/156)
**CA125, median (range), U/ml**	22.1 (5–736.2)
Elevated	31.9% (46/144)
Stage I/II	11.1% (16/144)
Stage III/IV	20.8% (30/144)
Normal	68.1% (98/144)
**FIGO stage**	
Stage I	53.2% (83/156)
Stage II	5.8% (9/156)
Stage III	29.5% (46/156)
Stage IV	11.5% (18/156)
**Rate of pathology coincidence**	
Hysteroscopy	69.6% (32/87)
Non-hysteroscopy	59.1% (55/87)
**Histology, No. (%)**	
Pure CCC	59% (92/156)
Mixed CCC	41% (64/156)
CCC + EmC	52
CCC + EmC + carcinosarcoma	1
CCC + EmC + SC	3
CCC + SC	8
**LVSI**	
( +)	25% (39/156)
**MI**	
≥ 1/2	40.6% (63/155)
**PWC**	
( +)	14.9% (20/134)
**Lymph node metastasis**	
No	70.2% (99/141)
Yes	29.8% (42/141)
PLN ( +) PALN ( −)	25
PLN ( −) PALN ( +)	6
PLN ( +) PALN ( +)	11
**Adjuvant treatment**	83.3% (130/156)
CT	81
RT	5
CRT	44
**No. of postoperative chemotherapy cycles, median (range)**	5 (1–9)

*CA125* Cancer antigen 125, *CCC* Clear cell carcinoma, *EmC* Endometrioid carcinoma, *SC* Serous carcinoma, *LVSI* Lymphovascular space invasion, *MI* Myometrial invasion, *PWC* Peritoneal washing cytology, *CT* Chemotherapy, *RT* Radiation therapy, *CRT* Chemoradiation, *PLN* Pelvic lymph nodes, *PALN* Para-aortic lymph nodes

### Surgical treatment

In terms of surgical approach, 124 patients (79.5%) underwent laparotomy, while 32 patients (20.5%) underwent laparoscopic surgery. All patients underwent TH and 99.4% (155/156) underwent BSO. A 31-year-old patient with stage IA disease preferred to undergo bilateral salpingectomy (BS) because of a strong desire to preserve reproductive potential. A few patients (*n* = 15) did not undergo lymph node evaluation: 10 patients had stage I disease, two had stage II disease, one had stage IIIb disease, and two had IVb disease. The remaining 141 patients underwent nodal assessment and approximately two-thirds underwent pelvic and para-aortic lymphadenectomies. Optimal cytoreductive surgery was performed in 153 (98.1%) patients. The details of the surgical treatments are presented in Table 2.

**Table 2:** Details of surgical procedures performed

**Surgical routes**	
Laparotomy	79.5% (124/156)
Laparoscopy	20.5% (32/156)
**Optimal cytoreduction**	
Yes	98.1% (153/156)
R0	140
R1	13
No	1.9% (3/156)
**Hysterectomy**	
Extrafascial hysterectomy	89.7% (140/156)
Radical hysterectomy	4.5% (7/156)
Modified radical hysterectomy	5.8% (9/156)
**Adnexectomy**	
BSO	99.4% (155/156)
BS	0.6% (1/156)
**Lymphadenectomy**	90.4% (141/156)
Pelvic alone	33.3% (47/141)
Pelvic + para-arotic	66.0% (93/141)
Unknown	1
**Harvested number of lymph nodes, median (range)**	30 (1–86)
PLN	24 (1–64)
PALN	6 (1–39)
Other	3 (1–13)
**Omentectomy**	85.3% (133/156)
Complete resection	130
Biopsy or partial resection	3
**Other surgical procedures**	96
**Appendectomy**	79^a^
**Intestinal resection and anastomosis**	2
**Other cytoreductive procedures**	15

*BSO* Bilateral salpingo-oophorectomy, *BS* Bilateral salpingectomy, *PLN* Pelvic lymph nodes, *PALN* Para-aortic lymph nodes; ^a^ There were another 16 patients with a history of appendectomy

### Histological diagnosis

Dilation and curettage, and hysteroscopy are the most common approaches used for preoperative diagnostic endometrial sampling. The concordance between the preoperative and postoperative histological results was higher in the hysteroscopy group (69.6%) than in the non-hysteroscopy group (59.1%) (Table 1). However, this difference was not statistically significant (*p* = 0.23). In terms of the histological type, 92 (59%) patients were pathologically diagnosed with pure ECCC, whereas 64 patients had MEC. Of the 141 patients who underwent nodal assessment, only 29.8% had histologically confirmed positive lymph nodes. Of the 42 patients with metastatic lymph nodes, 36 had pelvic lymph node metastasis with or without para-aortic lymph node metastasis, while six patients had isolated para-aortic nodal metastasis (Table 1).

### Relationship between clinicopathologic features and FIGO stage

Table 3 shows the relationship between clinicopathological features and FIGO stage. Overall, 92 (59%) patients had early-stage (stage I–II) disease, whereas 64 (41%) had advanced-stage (stage III–IV) disease. The serum CA125 level was significantly higher in patients with advanced-stage disease compared with the levels in those with early-stage disease (89.96 ± 146.08 vs 38.98 ± 75.74 IU/mL, *p* < 0.001). Additionally, patients with advanced-stage disease were more likely to have mixed pathological components than those with early-stage disease (54.7% vs. 31.5%; *p* = 0.004). Elevated CA125 levels (50% vs. 19.0%, *p* < 0.001), positive lymphovascular space invasion (LVSI) (46.9% vs. 9.8%, *p* < 0.001), deep myometrial invasion (MI) (66.7% vs. 22.8%, *p* < 0.001), and positive peritoneal washing cytology (PWC) (31.4% vs. 4.8%, *p* < 0.001) were more frequently observed in patients with advanced-stage ECCC than in patients with early-stage ECCC.

**Table 3:** The relationship between clinicopathological features and the FIGO stage

**Variate**	**Early (I/II) 59%, 92/156**	**Advanced (III/IV) 41%, 64/156**	***P*** ** value**
**Age, mean ± SD, y**	60.16 ± 10.99	61.98 ± 10.27	0.297
**Menopause, No. (%)**			
**No**	22 (23.9%)	8 (12.5%)	0.075
**Yes**	70 (76.1%)	56 (87.5%)	
**CA125, mean ± SD, U/ml**	38.98 ± 75.74	89.96 ± 146.08	< 0.001
** Normal**	68 (81.0%)	30 (50.0%)	< 0.001
** Elevated**	16 (19.0%)	30 (50.0%)	
**Histology, No. (%)**			
**Mixed**	29 (31.5%)	35 (54.7%)	0.004
**Pure**	63 (68.5%)	29 (45.3%)	
**LVSI, No. (%)**			
**(-)**	83 (90.2%)	34 (53.1%)	< 0.001
**( +)**	9 (9.8%)	30 (46.9%)	
**MI, No. (%)**			
** < 1/2**	71 (77.2%)	21 (33.3%)	< 0.001
** ≥ 1/2**	21 (22.8%)	42 (66.7%)	
**PWC, No. (%)**			
**(-)**	79 (95.2%)	35 (68.6%)	< 0.001
**( +)**	4 (4.8%)	16 (31.4%)	
**No. of postoperative chemotherapy cycles, mean ± SD**	3.94 ± 2.02	4.71 ± 1.93	0.039
**Recurrence, No. (%)**			
**No**	81 (88.0%)	36 (56.3%)	< 0.001
**Yes**	11 (12.0%)	28 (43.7%)	
**Mean time to recurrence, mean ± SD, months**	30.24 ± 30.33	11.57 ± 9.12	0.001
**Death, No. (%)**			
**No**	80 (87%)	44 (68.8%)	0.006
**Yes**	12 (13%)	20 (31.2%)	
**Mean time to death, mean ± SD, months**	64.00 ± 68.84	26.50 ± 21.07	0.024

*CA125* Cancer antigen 125, *LVSI* Lymphovascular space invasion, *MI* Myometrial invasion, *PWC* Peritoneal washing cytology

### Post-surgical adjuvant treatment

Postoperative adjuvant treatment was administered to 130 patients (83.3%), including CT in 81, RT in 5, and CRT in 44 (Table 1). Among patients who underwent CT with or without RT (125/156, 80.1%), 98.4% received platinum-containing regimens, and carboplatin/paclitaxel (TC) was the most frequently used chemotherapeutic regimen (78.4%). The median number of postoperative CT cycles was five (range, 1–9) (Table 1). Patients with advanced-stage ECCC were prone to receive more cycles of CT than those with early-stage ECCC (4.71 ± 1.93 vs. 3.94 ± 2.02, *p* = 0.039). A total of 24 patients did not receive any adjuvant therapy, including 17 patients with stage I, 3 with stage II, 3 with stage III, and 1 with stage IV ECCC. Two patients with stage IA disease underwent postoperative hormone therapy.

### Clinicopathologic factors associated with recurrence and survival outcomes

With a 69-month follow-up period (95% CI:51.24–86.50), 39 patients (25%) experienced relapses, while 32 patients (20.5%) died. Of the 39 patients who experienced recurrence, recurrence sites were identified in 27. The most common sites of recurrence among these patients were the lungs, retroperitoneal lymph nodes, abdomen, spleen, vaginal stump, perineum, bowel, brain, pelvis, liver, gallbladder, and skeleton. Eleven of 27 patients (40.7%) had multiple metastases. The frequencies of recurrence and death were significantly different between early and advanced stages (*p* < 0.001 and *p* = 0.006, respectively). The mean times to recurrence (30.24 ± 30.33 vs. 11.57 ± 9.12, *p* = 0.001) and death (64 ± 68.84 vs. 26.5 ± 21.07, *p* = 0.024) in patients with an early-stage disease were significantly longer compared with those in patients with an advanced-stage disease (Table 3).

**Fig. 2:**
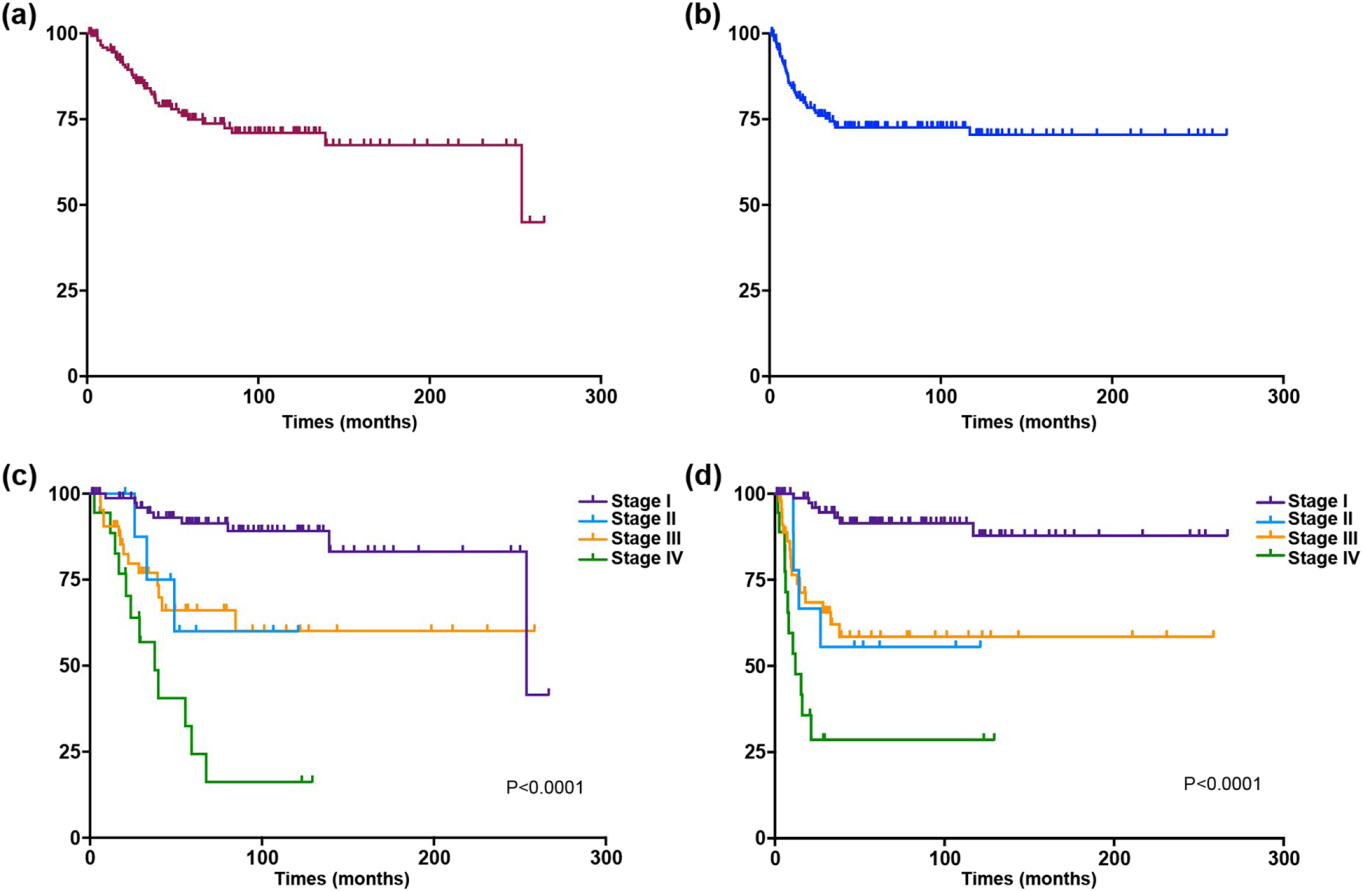
The Kaplan–Meier curves for the PFS and OS. Kaplan-Meier curves of 156 patients with ECCC. **A** OS of the entire cohort. **B** PFS for the entire cohort. **C** OS according to stage. **D** PFS according to stage. OS, overall survival. PFS, progression-free survival

The 5-year PFS and OS rates for the entire cohort were 72.6% and 79%, respectively. Further analysis revealed that in patients with FIGO stages I–IV, the 5-year PFS and OS rates were 91.5% and 91.4%; 55.6% and 60%; 58.5% and 66.1%; and 28.6% and 55.6%, respectively. Figure 2 illustrates the Kaplan–Meier curves for PFS and OS of the entire cohort. Both PFS and OS were significantly associated with disease stage (*p* < 0.0001). Univariate and multivariate Cox regression analyses were performed to delineate prognostic factors for PFS and OS (Table 4). In the univariate analysis, elevated CA125 levels (*p* = 0.012), advanced-stage disease (*p* < 0.001), presence of LVSI (*p* = 0.026), and positive PWC were associated with worse PFS (*p* < 0.05). However, optimal cytoreduction was a favorable prognostic factor for PFS (HR:0.18 [95% CI:0.04–0.76], *p* = 0.019). The multivariable analysis demonstrated a significant decrease in PFS in patients with advanced-stage disease (HR:5.95 [95% CI:2.26–15.68], *p* < 0.001) and positive PWC (HR:3.27 [95% CI:1.43–7.49], *p* = 0.005). Similarly, older age (> 61 years) (*p* = 0.006), advanced-stage disease (*p* = 0.001), presence of LVSI (*p* = 0.04), deep MI (≥ 1/2) (*p* = 0.015), and positive PWC (*p* = 0.025) had a significantly poor impact on OS in the univariate analysis. Elevated CA125 levels (*P* = 0.05) had a borderline effect on OS. When the aforementioned parameters were included in a multivariable model, only age > 61 years (HR:4.30 [95% CI:1.51–12.24], *p* = 0.006) and advanced-stage disease (HR:3.96 [95% CI:1.39–11.24], *p* = 0.01) were associated with decreased OS.

**Table 4:** The prognostic factors for PFS and OS on univariate and multivariate Cox regression analyses

**Variables**	**PFS**				**OS**			
	**Univariate**		**Multivariate**		**Univariate**		**Multivariate**	
	HR (95%CI)	***P*** ** value**	HR (95% CI)	***P*** ** value**	HR (95%CI)	***P*** ** value**	HR (95% CI)	***P*** ** value**
**Age at diagnosis, years**								
** ≤ 61**	Reference				Reference			
** > 61**	1.805 (0.946–3.443)	0.073			2.985 (1.372–6.491)	0.006	4.297 (1.509–12.238)	0.006
**CA125**								
**Normal**	Reference				Reference			
**Elevated**	2.357 (1.208–4.599)	0.012			2.121 (1.001–4.495)	0.050		
**FIGO Stage**								
**I/II**	Reference				Reference			
**III/IV**	5.501 (2.725–11.103)	0.000	5.947 (2.256–15.678)	0.000	3.545 (1.725–7.288)	0.001	3.958 (1.394–11.238)	0.010
**Cytoreduction**								
**Sub-optimal**	Reference				Reference			
**Optimal**	0.179 (0.042–0.756)	0.019			0.502 (0.068–3.694)	0.499		
**Histology**								
**Mixed**	Reference				Reference			
**Pure**	1.939 (0.965–3.895)	0.063			1.293 (0.63–2.65)	0.483		
**LVSI**								
**(-)**	Reference				Reference			
**( +)**	2.108 (1.094–4.062)	0.026			2.12 (1.034–4.346)	0.040		
**MI**								
** < 1/2**	Reference				Reference			
** ≥ 1/2**	1.806 (0.954–3.419)	0.069			2.409 (1.19–4.878)	0.015		
**PWC**								
**(-)**	Reference				Reference			
**( +)**	5.737 (2.662–12.363)	0.000	3.27 (1.428–7.49)	0.005	2.935 (1.146–7.513)	0.025		

*CI* Confidence interval, *PFS* Progression-free survival, *OS* Overall survival, *CA125* Cancer antigen 125, *LVSI* Lymphovascular space invasion, *MI* Myometrial invasion, *PWC* Peritoneal washing cytology

## Discussion

In total, 156 patients with ECCC were included in our analysis, consisting of patients with pure ECCC (*n* = 92) and MEC with CCC components (*n* = 64). At a median follow-up of 5.8 years, the recurrence and death rates in our cohort were 25 and 20.5%, respectively. Long-term follow-up showed that the 5-year OS rate of the entire cohort was 79%, which was significantly influenced by the disease stage. In general, our cohort had a good prognosis compared with those evaluated in other studies [11–14]. Patients with MEC have a worse prognosis than those with pure high-grade carcinomas [15].

Compared to endometrioid carcinoma, type II EC and the presence of type II components are usually correlated with more aggressive tumor features, including age of onset, FIGO stage, adnexal and cervical invasion, and extrauterine metastasis. ECCC is an infrequent type II EC usually diagnosed at an advanced stage of the disease. Early diagnosis, and timely and prompt treatment can improve cancer prognosis. Early screening is one of the greatest challenges in cancer research. In recent years, metabolomics has become a promising approach for uncovering the underlying mechanisms of EC. Metabolomics analysis can be used for the early detection of EC. Troisi et al. [16] validated a metabolomics-based algorithm for screening EC and illustrated that the serum metabolome is a promising screening test for EC because of its excellent performance. Because of the aggressive behavior of ECCC, we hope that more studies will be conducted to explore the relationship between ECCC and metabolomics, which could have an extraordinary impact on the management of ECCC in the future.

In our cohort, the median age was 61 years (range, 31–6 years) and 41% of the patients were diagnosed with advanced-stage ECCC. In the present study, 80.8% of women were postmenopausal. Chao et al. found that elevated serum CA125 levels usually indicate deep MI, extrauterine spread, positive PWC, lymph node metastasis, advanced-stage disease, and recurrence [17]. In line with the literature [18], one-third of the patients (46/144, 31.9%) in our cohort had elevated serum CA125 levels and 30 of 46 (65.2%) patients were diagnosed with stage III–IV disease. Compared to patients with early-stage disease, those with advanced-stage disease had a higher prevalence of positive LVSI, mixed histology, deep MI, positive PWC, and elevated serum CA125 levels (*p* < 0.05). However, Cetinkaya et al. found that the prevalence of advanced-stage disease was significantly higher in patients with pure ECCC than in those with mixed histological subtypes (*p* = 0.04) [18]. These results contradict the findings of this study. First, only 26 patients with pure ECCC or a mixed histology were included. Mixed ECCC was defined as the presence of at least 50% clear cell components.

In our study, 41% of patients had MEC with CCC components. In addition to CCC, EMC and SC are the two most commonly encountered histological components. In the analysis by Murphy et al., 47% of patients were classified as having a pure clear cell type. Of the 20 patients with MEC with CCC components, the other two distinctive components were EmC and SC [19]. Increasing evidence has shown that ECCC has a substantial overlap with both SC and EmC, not only in terms of morphology and immunophenotype, but also in terms of molecular characterization [20]. Bae et al. [21] evaluated the clinicopathological, immunohistochemical, and molecular features of ECCC and found that pure ECCC had immunohistochemical and molecular characteristics similar to those of EmC and SC. Matrai et al. evaluated eight patients with MEC (four with EmC + SC, one with SC + CCC, and three with EmC + CCC) to examine their underlying molecular features and oncogenic mechanisms. Their data suggested that all tumors shared mutations in both components and that the majority of these tumors originated from a single clone with subsequent divergence [13, 20]. This may explain why EmC and SC are the most common admixtures of mixed ECCC.

Almost all patients in our cohort underwent THBSO, of whom 90.4% underwent nodal assessment and 85.3% underwent omental biopsy or omentectomy. The reasons for incomplete surgical staging included medical comorbidities, older age, postoperative incidental diagnosis of ECCC, surgeon discretion, and unknown reasons for external surgery. A total of 130 patients received adjuvant therapy, and another two patients received hormone therapy. Previous studies examining the benefits of adjuvant treatment [7, 9, 19, 22–28] for ECCC have reported conflicting results. Previous studies have reported conflicting and contradictory conclusions regarding the effects of adjuvant treatment, possibly because the details of CT and RT, including regimen, field, dose, and fractionation, were inconsistent. Notably, whether adjuvant therapy should be administered usually depends on the stage and clinicopathological factors. In contrast, high-risk factors and individual variations differed significantly among patients at the same stage. Therefore, research on the effects of adjuvant therapy on survival outcomes is only considered significant if it is conducted on appropriately selected patients, such as those with the same stage (or even substages) and high-risk factors. However, given the low event rate of this rare neoplasm, it is difficult to evaluate a large cohort of patients with ECCC and divide them into subgroups of the same stage and risk. Considering these factors, the differences in oncological outcomes among the different postoperative adjuvant treatments were not assessed in our analysis. According to the National Comprehensive Cancer Network clinical practice guidelines for uterine neoplasms (version 1.2022) [29], TC is the preferred regimen for patients with uterine-defined, high-risk disease, and recurrent or metastatic disease, regardless of carcinoma histology. In line with the literature and the National Comprehensive Cancer Network clinical practice guidelines, postoperative adjuvant CT was administered to 80.1% of the patients in our cohort, and TC was used as the first-line treatment. TC combined with RT appears to be effective, less toxic, and well tolerated in the management of patients with ECCC [9].

In our study, PFS and OS were significantly influenced by the tumor stage (*p* < 0.001). Patients in the advanced stage had a higher recurrence rate (43.7% vs. 12%, *p* < 0.001) and overall death rate (31.2% vs. 13%, *p* = 0.006); the mean times to recurrence (11.57 ± 9.12 vs. 30.24 ± 30.33, *p* = 0.001) and death (26.50 ± 21.07 vs. 64.00 ± 68.84, *p* = 0.024) were also significantly shorter. We reviewed the current literature regarding prognostic factors for ECCC survival and found that LVSI [22, 26–28, 30, 31], disease stage [31–33], age [33], PWC [13, 27, 34], and MI [22, 31, 32, 35] were correlated with ECCC prognosis. Notably, both ECCC and serious uterine carcinoma have been analyzed in most of these studies. Our study included only patients with ECCC. In the univariate analysis, we found that elevated CA125 levels, optimal cytoreductive surgery, LVSI, positive PWC, and advanced stage were prognostic factors for PFS. After adjusting for these clinicopathological features, only advanced-stage disease and positive PWC were significantly associated with a poor PFS. Similarly, LVSI, positive PWC, advanced-stage disease, older age (> 61 years), and deep MI were prognostic factors for OS in the univariate analysis. However, multivariate analysis showed that only older age (> 61 years) and advanced disease stage were independent prognostic factors for OS. In 2013, The Cancer Genome Atlas (TCGA) demonstrated that EC can be divided into four molecular groups [36]. Notably, the integrated genomic characterization of EC was performed in patients with EmC and SC. In 2020, a novel risk stratification model [37] that recommended the integration of the TCGA molecular signature with classic pathological factors to assess the prognosis of EC was proposed. However, little is known about the molecular features of ECCC. Therefore, pathological factors remain the classic prognostic factors for patients with ECCC. Some studies [38, 39] have analyzed the relationship between TCGA groups and classic prognostic factors such as MI and LVSI. Raffone et al. [39] conducted a systematic review and meta-analysis and found that the prognostic value of LVSI, age, and adjuvant treatment was independent of the TCGA classification. LVSI correlates with a 1.5 to 2 times risk of death due to any cause, EC-related death, recurrence and progression [39].

The significance of PWC has been debated over recent decades. Prior to the publication of FIGO 2009 classification, positive PWC were used as a variable to define the stage. However, subsequent studies of the significance of PWC have yielded conflicting results. Additionally, the role of PWC in ECCC and other types of type II EC has not been individually evaluated [13]. Finally, positive PWC were removed from the FIGO 2009 stage matrix [10]. Peritoneal cytology does not affect staging, but should be performed during THBSO [10, 29]. Consistent with the findings of other studies [13, 40], our data also demonstrated that positive PWC were an independent negative prognostic factor for PFS in patients with ECCC, even though the presence of PWC had no influence on disease stage. This suggests that obtaining peritoneal cytology samples during THBSO is essential.

To the best of our knowledge, the current study is the largest Chinese study to examine the clinicopathological features and survival outcomes of 156 patients with ECCC. Overall, our study had an adequate follow-up time of 69 months compared with other similar studies. To minimize selection bias, we enrolled patients in a 35-year time span. In addition, objective indicators were used to collect clinical information, and the study population was selected strictly according to the inclusion and exclusion criteria. Finally, in our department, all patients with EC were regularly followed up through telephone calls after completing cancer-related treatment. This can improve the compliance of patients and their families and reduce the non-response rate caused by missing visits as much as possible. However, the retrospective design has some inherent unmeasured bias. Our study has some limitations. Another limitation of our study was that 59% of the patients had pure ECCC, which is not sufficient to broadly represent the ECCC population. The remaining 41% of the cohort had MEC, and the pathology reports did not show the relative proportions of each component. Finally, owing to the retrospective nature of the study, the peritoneal cytological results of 22 patients were unavailable. Therefore, we concluded that peritoneal washing was not performed in these patients. These limitations limited the power of our study.

To fully understand the oncological outcomes and prognostic factors of ECCC, large-scale prospective trials should be conducted globally to include a multiracial and representative study group and the results should be considered more reliable. However, owing to the rarity and aggressive nature of ECCC, such studies are difficult to conduct. The present study adds to the growing body of evidence to fill this gap in the Chinese population. We hope that this study will provide physicians and scientific workers with valuable data regarding patients with ECCC.

## Conclusions

ECCC is a rare type of EC that is clinically aggressive and common in older women and those with advanced-stage disease. Positive PWC were associated with poorer PFS, although the presence of PWC did not influence the disease stage. Positive PWC, advanced stage, and older age were independent negative prognostic factors for ECCC.

## Data Availability

The datasets used and/or analyzed in the current study are available from the corresponding author upon reasonable request.

## Abbreviations

ECCC: Endometrial clear cell carcinoma
OS: Overall survival
PFS: Progression-free survival
CCC: Clear cell carcinoma
PWC: Peritoneal washing cytology
EC: Endometrial carcinoma
MEC: Mixed endometrial carcinoma
EmC: Endometrioid cancer
SC: Serous carcinoma
THBSO: Total hysterectomy and bilateral salpingo-oophorectomy
TH: Total hysterectomy
BSO: Bilateral salpingo-oophorectomy
FIGO: International Federation of Gynecology and Obstetrics
CT: Chemotherapy
RT: Radiation therapy
CRT: Chemoradiation
CA125: Cancer antigen 125
CI: Confidence interval
BS: Bilateral salpingectomy
LVSI: Lymphovascular space invasion
MI: Myometrial invasion
TC: Carboplatin/paclitaxel
